# Maturity Related Differences in Body Composition Assessed by Classic and Specific Bioimpedance Vector Analysis among Male Elite Youth Soccer Players

**DOI:** 10.3390/ijerph17030729

**Published:** 2020-01-22

**Authors:** Stefania Toselli, Elisabetta Marini, Pasqualino Maietta Latessa, Luca Benedetti, Francesco Campa

**Affiliations:** 1 Departments of Biomedical and Neuromotor Sciences, University of Bologna, 40121 Bologna, Italy; stefania.toselli@unibo.it; 2 Department of Life and Environmental Sciences, Neuroscience and Anthropology Section, University of Cagliari, Monserrato, 09042 Cagliari, Italy; emarini@unica.it; 3 Department for Life Quality Studies, University of Bologna, 47921 Rimini, Italy; pasqualino.maietta@unibo.it; 4 School of Pharmacy, Biotechnology and Sport Science, University of Bologna, 40126 Bologna, Italy; lucaben759@gmail.com

**Keywords:** age at peak height velocity, BIVA, phase angle, R-Xc graph

## Abstract

The aim of this study was to analyze the efficiency of classic and specific bioelectrical impedance vector analysis (BIVA) in the assessment of maturity related differences in body composition among male elite youth soccer players, and to provide bioelectrical impedance reference data for this category. A group of 178 players (aged 12.1 ± 1.6 years) were registered in a professional Italian soccer team participating in the first division (Serie A). They were divided into three groups according to their maturity status while bioelectrical resistance and reactance were obtained. The classic and specific BIVA procedures were applied, which correct bioelectrical values for body height and body geometry, respectively. Percentage of fat mass (FM%) and total body water (TBW (L)) were estimated from bioelectrical values. Age-specific z-scores of the predicted age at peak height velocity identified 29 players as earlier-, 126 as on time-, and 23 as later-maturing. TBW was higher (*p* < 0.01) in adolescents classified as “early” maturity status compared to the other two groups and classic BIVA confirmed these results. Conversely, no differences in FM% were found among the groups. Specific vector length showed a higher correlation (*r* = 0.748) with FM% compared with the classic approach (*r* = 0.493). Classic vector length showed a stronger association (*r* = −0.955) with TBW compared with specific (*r* = −0.263). Specific BIVA turns out to be accurate for the analysis of FM% in athletes, while classic BIVA shows to be a valid approach to evaluate TBW. An original data set of bioelectric impedance reference values of male elite youth soccer players was provided.

## 1. Introduction

Analyzing and monitoring body composition (BC) is an important topic in sports because of its influence on health status and physical performance [1,2,3]. In fact, an elevated fat mass percentage (FM%) negatively influences the quality of functional movement patterns, reducing physical performance in athletes [4,5,6]. Conversely, wider muscle mass areas, particularly of the upper limb, maximizes power expression and the capacity to perform repeated sprints with changes in direction [7,8].

An interesting approach commonly used in the sports field to evaluate BC is bioelectrical impedance vector analysis (BIVA) [9]. This method plots the impedance parameters on a graph as a single vector. Classic BIVA is the traditional vector method proposed by Piccoli et al. [10], where bioimpedance measurements (resistance (R) and reactance (Xc)) are adjusted for the height of the subjects. Changes in vector length represent variations in total body water (TBW) or in FM% depending on which BIVA approach is applied [11,12,13,14,15,16]. The specific BIVA method corrects R and Xc according to the body cross-sections in addition to height [17]. The vector slope in the R-Xc graph correctly identifies changes in the intracellular/extracellular water (ICW/ECW) ratio in both classic and specific BIVA. The ICW/ECW ratio is also represented by the phase angle (PA) which is calculated by the arctangent of Xc/R × 180°/π and hence is the same in the two approaches [18,19].

An influencing factor of BC is somatic maturation [20,21]. Athletes with similar chronological age competing in the same category levels can, in fact, show a difference in maturity status, where the offset is reached at 14 ± 1 years in boys and at 12 ± 1 years in girls [22]. Subjects in early maturation show higher muscle mass and body dimensions compared with peers in late maturation, which also implies a better physical performance [23,24,25]. Moreover, Koury et al. [26] suggested that maturity status is influenced by bioimpedance values in young male soccer athletes, highlighting the differences in BC between non-mature and mature male adolescent soccer players.

Marini et al. [27] provided solid evidence regarding the accuracy of classic and specific BIVA in the assessment of BC in athletes. Based on the comparison with reference techniques (dual X–ray absorptiometry (DXA) and dilution methods), they showed that specific BIVA is more accurate in the FM% evaluation, whereas it does not correctly evaluate TBW, for which classic BIVA appears to be a suitable method, and that PA can detect ECW/ICW changes in both approaches [27]. However, no other study has been carried out to confirm this thesis on an athlete sample, assessing the effects that differences in maturity status may have on BIVA patterns. In addition, while bioimpedance standards have been established for the normal healthy population or in the clinical setting, they are not available for male elite youth soccer players, nor in many other sports [28,29]. Bioimpedance reference data allows for the creation of tolerance ellipses on the R-Xc graphs for ranking body fluid contents and ICW/ECW ratio. These ellipses, specifically for age and sports categories, assist in the BC evaluation without using prediction equations [9]. Furthermore, evaluating BIVA patterns within the R-Xc graph can provide important information regarding the somatic maturation of the subjects [20]. For this reason, it is necessary to collect new data on the BIVA vector changes among subjects with different maturity statuses.

Therefore, the main purpose of the present study was to assess BC using classic and specific BIVA, identifying the differences in BC, particularly in TBW (classic BIVA) and in FM% (specific BIVA), due to the influence of somatic maturation. The second aim was to provide specific R-Xc graphs for male elite youth soccer players. We hypothesized that BIVA patterns are differentiated among athletes according to whether or not they mature earlier, on-time, or late.

## 2. Materials and Methods

### 2.1. Subjects

The study included 178 elite-level male youth soccer players (age 12.1 ± 1.6 years), from the U10–U15 age categories (U10, *N* = 26; U11, *N* = 26; U12, *N* = 26; U13, *N* = 41; U14, *N* = 31; U15, *N* = 28), associated with an Italian Serie A professional soccer team. The eligibility criteria for this study were that the players would be free from injury and illness during the research analyses. The players voluntarily decided to participate, and their parents provided informed consent after a detailed description of the study procedures. The study was carried out in conformity with the ethical standards laid down in the 1975 declaration of Helsinki and was approved by the local Bioethics Committee of the University of Bologna (Ethical Approval Code: 25027; dated 13 March 2017).

### 2.2. Procedures

Bioimpedance and anthropometric data were collected at the site of the youth academy. The testing procedures were performed during the first part of the preparation period of the 2019–2020 season (August 2019), after a recovery microcycle. The participants had a balanced breakfast 4 h before the measurements, which were held in the morning (11:00 a.m.).

The impedance measurements were performed with bioimpedance analysis (BIA 101 Anniversary, Akern, Florence, Italy) using an electric current at a frequency of 50 kHz. The subjects were in the supine position with a leg opening of 45° compared to the median line of the body and the upper limbs positioned 30° away from the trunk. After cleansing the skin with alcohol, two Ag/AgCl low-impedance electrodes (Biatrodes, Akern Srl, Florence, Italy) were placed on the back of the right hand and two electrodes on the corresponding foot, with a distance of 5 cm between each other [30]. Bioelectrical impedance vector analysis was carried out using the classic and specific BIVA methods, i.e., normalizing resistance and reactance for height in meters (classic BIVA [14]), or multiplying R and Xc by length and cross-sectional measures (specific BIVA [15]). Impedance was calculated as (adjusted R^2^ + adjusted Xc^2^)^0.5^ and phase angle (PA) as the arctangent of Xc/R × 180°/π. FM, fat-free mass (FFM), and TBW were predicted using Bodygram PLUS Software V. 1.0 (Akern Srl., Pontassieve, Florence, Italy).

Height and sitting height were measured to the nearest 0.1 cm using an anthropometer (Raven Equipment Ltd, Great Donmow, UK). Each player’s leg length was calculated as the difference between their recorded body height and sitting height. Body mass was measured to the nearest 0.1 kg using calibrated electronic scales (Seca, Basel, Switzerland). An estimation of the years from peak height velocity, which is an indicator for the adolescent growth spurt, was made using the Mirwald equation for boys, which is able to predict maturity offset in youth athletes [31,32].

Maturity offset = −9.236 + 0.0002708 (leg length ∗ sitting height) − 0.001663 (age ∗ leg length) + 0.007216 (age ∗ sitting height) + 0.02292 (weight: height).

Since Maturity offset represents the time before or after peak height velocity (PHV), years from PHV were calculated by subtracting age at PHV from chronological age. To classify players according to their maturity status, we followed the approach proposed by Rommers et al. [24], who overcame the age effect by using z-scores. Age at peak height velocity (APHV) z-scores of the predicted APHV were calculated within each age category (U10–U15, N = 6) and used to classify players as “earlier” (z < −1), “on-time” (−1 ≤ z ≤ 1), or “later” (z > 1) maturing.

### 2.3. Statistical Analysis

Descriptive statistics including means (SD) were calculated for all outcome variables. To verify the normality of the data, the Shapiro–Wilk test was applied. Univariate analysis of variance for multiple comparisons was performed. When a significant F ratio was obtained, the Bonferroni post-hoc test was used to assess the differences among the groups. A semi-quantitative bioelectrical analysis was realized using tolerance ellipses that represent the bioelectrical variability of the reference population. The upper pole, defined by longer impedance values, is indicative of lower TBW in classic BIVA, and higher FM% in specific BIVA; the left side, defined by higher PA values, is indicative of a higher ICW/ECW ratio in both BIVA approaches [7,27]. The two-sample Hotelling’s T^2^ test was used to compare the mean impedance vectors of the different groups; separate 95% confidence ellipses indicated a significant vector difference. The association between bioelectrical impedance and TBW and FM% values was investigated using Pearson’s correlation analysis. Statistical significance was pre-determined as *p* < 0.05. SPSS (23.0.0.0; SPSS Inc., Chicago, IL, USA) was used for all statistical calculations.

## 3. Results

Due to the maturity status categorization based on normally-distributed age-specific z-scores, 70.8% of the players were classified as “on-time”, 16.3% as “earlier”, and 12.9% as “later” maturing, within each age category.

Height, sitting height, weight, as well as BMI were higher (*p* < 0.025) in adolescents classified as “early” maturity status compared with the other two groups. In addition, early- and on-time-maturing athletes showed an earlier APHV with respect to the late-maturing athletes (Table 1).

**Figure 1:**
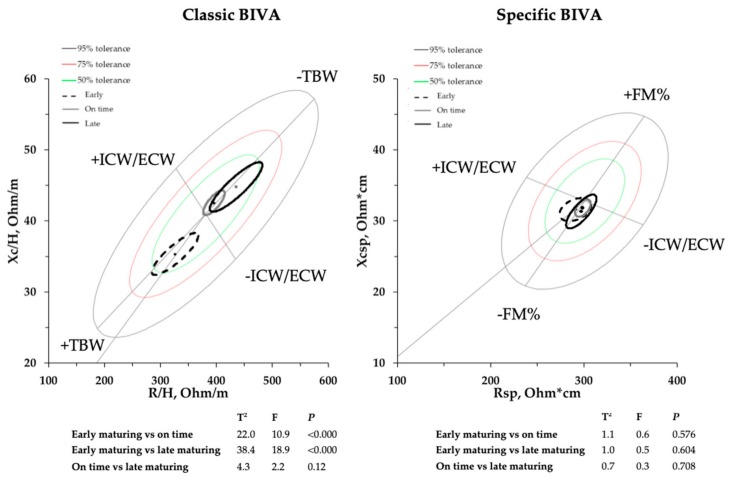
Main graph with 95% confidence ellipses plotted on the new reference ellipses; Hotelling’s T^2^ test results are included.

Early-maturing athletes showed significantly higher values for TBW, FFM, and FM with respect to the other groups, while FM% was similar (Table 2). Classic BIVA identified significant differences in “earlier” adolescents, while no differences were found by the specific BIVA approach among the soccer players with different maturity statuses (Figure 1). In fact, R/H and Xc/H showed significantly higher values with the increase of maturation-time, while Rsp, Xcsp, and PA did not change (Table 2). Measured bioimpedance values for the whole sample of athletes were R/H = 382.1 ± 81.6, Xc/H = 41.3 ± 7.8 and Rsp = 300.9 ± 35.9, Xsp = 32.8 ± 5.1, for classic and specific BIVA, respectively (Figure 1). In addition to the R/H and Xc/H values, the correlation coefficient r between R/H and Xc/H is needed to draw the ellipses. The correlation coefficients for r were r = 0.81 and r = 0.64, for classic and specific BIVA, respectively.

**Figure 2:**
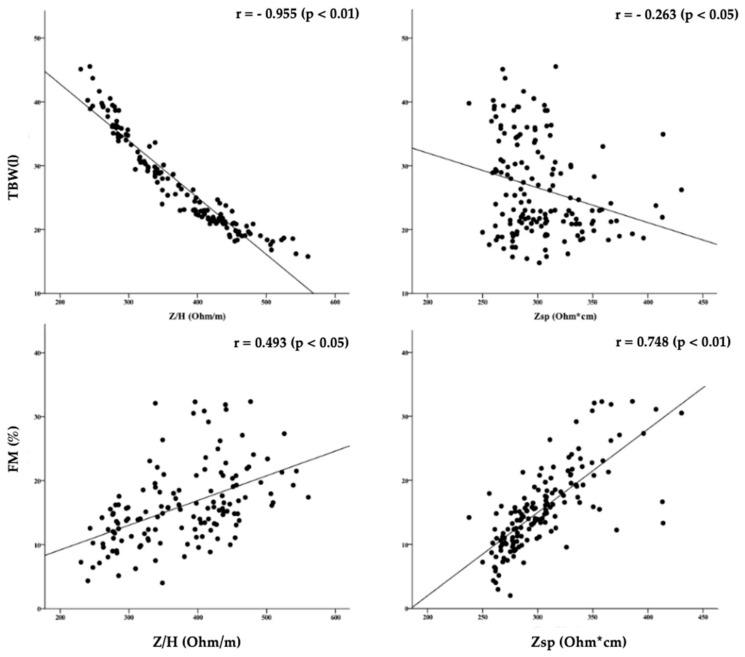
Correlation between classic or specific impedance vectors with TBW (L) or FM%.

The classic impedance vector (Z/H) was negatively associated with TBW (L), while the specific impedance vector (Zsp) was more strongly and positively associated with FM% (Figure 2).

**Figure 3:**
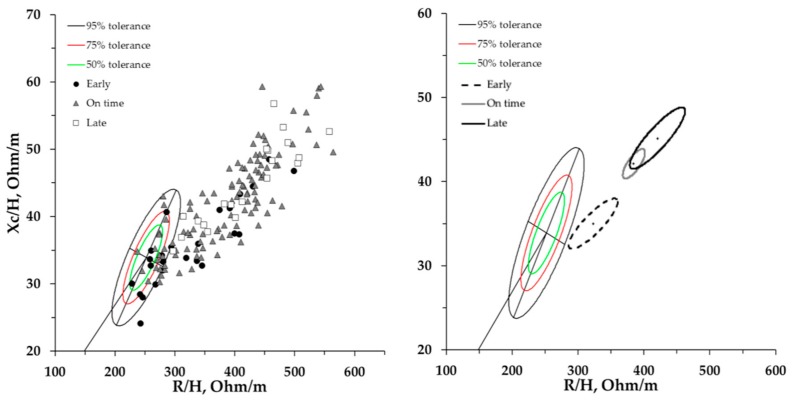
Mean and single impedance vectors plotted on the adult soccer players’ classic BIVA tolerance ellipses [29].

Figure 3 shows the single and mean impedance vectors of the athletes sorted based on maturity status in relation to the classic BIVA references for elite male soccer players [29].

## 4. Discussion

The present study compared classic and specific BIVA for BC assessment in male elite youth soccer players, with different maturity statuses. Athletes with an advanced maturity status showed higher TBW and a shorter classic vector length, while no differences in FM% or specific vectors were measured among the groups. There were no significant differences in PA, even though it is possible to note a tendency to increase with the advancement of somatic growth. Data confirmed the ability of specific BIVA in evaluating FM% and of the classic method in measuring TBW. Additionally, for the first time, new references for both classic and specific BIVA methods were provided for male elite youth soccer players. In this regard, although the classic BIVA references exist for some sports, specific BIVA references were lacking until now [33].

The main point of BIVA is to provide the classification of body fluids or FM% by comparing either different groups or the position of an individual vector to a reference population. For this reason, the application of both BIVA approaches can be compromised if non-appropriate reference R-Xc graphs are used, leading to evaluations that are difficult to interpret [33]. Differences between groups of athletes were consistently recognized by bioimpedance methods. In fact, earlier-maturing athletes showed shorter classic vector length, indicating higher TBW content, but a similar specific vector length, representing a similar FM%. In particular, Z/H (classic BIVA vector) showed significant associations with TBW, demonstrating the greater accuracy of this method in the evaluation of body fluids, while Zsp (specific BIVA) confirmed its limited sensitivity in the TBW evaluation. Conversely, Zsp showed a higher significant correlation with FM% compared to the classic approach. Unlike specific BIVA, the classic method is able to analyze body fluids with higher accuracy.

In this study, it was shown that growing athletes obtain specific BC characteristics. In this regard, it is well known that the morphology and BC features of athletes differ from the normal population, as well as within various sports and competitive levels [4,7,28,29,34,35]. The classic bioimpedance vector of the early-maturing athletes was shorter with respect to the other two groups, showing closer similarity to the BIVA patterns (e.g., vector length and PA) of elite adult soccer players. In particular, if single vectors are taken into consideration, a few early-maturing athletes are already within the 50th percentile of the adult soccer players’ R-Xc graph defined by Micheli et al. [29] (Figure 3). Furthermore, the mean vector displacement of the early-maturing athletes indicates that to achieve the BC soccer player profile, an increase in TBW and in the ICW/ECW ratio is necessary, therefore shortening the vector length and increasing the slope.

Previous studies have shown that vector length reduction is associated with growth in children, a trend that is accentuated with sexual maturation, as demonstrated by the bioelectrical differences among adolescents of similar age but different maturity status [33,36,37,38,39]. The results of this study suggest that the maturational development in elite athletes influences FFM and TBW, but not FM%. In this regard, it is possible that the scarce differences observed are to be interpreted in relation to the selection of these athletes. In line with these findings, Agostinete et al. [40] showed that the FM% is higher in young sedentary adolescents than in those who practice sports, without any impact from the maturity status. Moreover, previous studies have shown that FM% decreases with growth, showing a peak around 16 years, and then remaining constant [41,42].

Despite the encouraging results obtained in this study, some limitations are present and should be considered. In fact, our results are applicable to BIA equipment using the 50 kHz frequency and to a similar population. In addition, although showing a normal data distribution, the group of on-time-maturation athletes was much larger than the other two groups. Therefore, future studies should consider a larger sample size, also providing new BIVA references that consider different somatic maturation categories. Lastly, even if the Mirwald equations are widely used in the field of sport, it presents some limitations such as its dependence of predicted age at PHV upon chronological age at prediction and on actual age at PHV [43].

Due to the important implications of maturity status and BC on sports performance, future studies should identify BIVA reference values for each competitive category in young players. This would allow access to meaningful information on the maturity status of subjects, given its influence on BIVA patterns, through which it is possible to obtain an accurate evaluation of BC in athletes.

## 5. Conclusions

This study provides new 50%, 75%, and 95% classic and specific BIVA reference values for male elite youth soccer players. Physicians and coaches should consider using both BIVA approaches to obtain reliable BC evaluations, utilizing classic BIVA to evaluate changes in body fluids and specific BIVA to evaluate FM%. In this regard, TBW increases with the progression of somatic maturation, while FM% remains similar. Considering BIVA patterns, early-maturing athletes show a shorter vector length and a tendency to a greater PA than on-time- and late-maturing peers.

## Figures and Tables

**Table 1:** Results of ANOVA analyses and descriptive statistics of anthropometric features, according to maturity status.

Variable	Early-Maturing, *n* = 29	On-Time	Late-Maturing	F	*p*
		*n* = 126	*n* = 23		
Age, y	12.3 (1.6)	12.4 (1.6)	12.4 (1.7)	0.37	0.69
Height, cm	161.5 (24.8) # §	157.7 (35.8) *	149.6 (13.4) *	8.33	<0.001
Sitting height, cm	80.7 (13.2) # §	77.8 (16.9) *	76.7 (7.4) *	16.29	<0.001
Weight, kg	52.4 (27.6) # §	41.4 (28.5) *	37.2 (10.1) *	14.06	<0.001
BMI, kg/m^2^	19.5 (4.6) # §	19.2 (2.7) *	18.1 (1.7) *	9.41	<0.001
Predicted APHV, y	12.9 (0.3) # §	13.7 (0.1) * §	14.5 (0.3) * #	143.63	<0.001

Note: Data are presented as mean (SD). Abbreviations: ANOVA, analysis of variance; BMI, body mass index; APHV, age at peak height velocity. * Differences (*p* < 0.025) compared with the early-maturing group. # Differences compared with the on-time group. § Differences compared with the late-maturing group.

**Table 2:** Results of ANOVA analyses examining the differences in body composition and physical performance, according to maturity status.

Variable	Early-Maturing, *n* = 29	On-Time,	Late-Maturing,	F	*p*
		*n* = 126	*n* = 23		
TBW (L)	32.4 (8.3) # §	25.7 (6.8) *	22.9 (5.8) *	12.46	<0.001
FFM (kg)	45.1 (12.1) # §	35.6 (10.9) *	32.1 (9.4) *	11.06	<0.001
FM (kg)	7.3 (2.4) # §	5.8 (2.1) *	5.1 (1.7) *	7.52	0.001
FFM (%)	86.7 (4.1)	86.9 (3.8)	87.3 (2.9)	0.17	0.84
FM (%)	13.3 (4.1)	13.1 (3.8)	12.7 (2.9)	0.17	0.84
R/H (ohm/m)	325.3 (76.4) # §	387.4 (77.7) *	423.1 (75.3) *	9.99	<0.001
Xc/H (ohm/m)	35.7 (6.1) # §	41.9 (7.9) *	45.1 (6.2) *	10.22	<0.001
Rsp (ohm ∗ cm)	294.4 (34.3)	302.5 (37.7)	300.8 (28.8)	0.5	0.604
Xcsp (ohm ∗ cm)	32.7 (3.4)	32.9 (5.6)	32.4 (4.3)	0.11	0.889
PA (degrees)	6.4 (0.8)	6.3 (0.9)	6.2 (0.5)	0.46	0.631

Note: Data are presented as mean (SD). Abbreviations: ANOVA, analysis of variance; TBW, total body water; FFM, fat-free mass; FM, fat mass; R/H, resistance standardized for height; Xc/H, reactance standardized for height; Rsp, resistance standardized for height and transverse areas; Xcsp, reactance standardized for height and transverse areas; PA, phase angle. * Differences (*p* < 0.025) compared with the early-maturing group. # Differences compared with the on-time group. § Differences compared with the late-maturing group.
